# Construction and Application of a Model for Predicting the Risk of Delirium in Postoperative Patients With Type a Aortic Dissection

**DOI:** 10.3389/fsurg.2021.772675

**Published:** 2021-11-18

**Authors:** Junfeng He, Qing Ling, Yuhong Chen

**Affiliations:** ^1^Department of Nursing, Nanjing First Hospital, Nanjing Medical University, Nanjing, China; ^2^Department of Nursing, Nanjing First Hospital, Nanjing, China

**Keywords:** type A aortic dissection, postoperative delirium, predictive model, nursing, factor

## Abstract

**Background:** Postoperative delirium (POD), an alteration in a patient's consciousness pattern, can affect the treatment and prognosis of a disease.

**Objective:** To construct a prediction model for delirium in patients with type A aortic dissection after surgery and to validate its effectiveness.

**Methods:** A retrospective cohort design was used to study 438 patients undergoing surgical treatment for type A aortic dissection from April 2019 to June 2020 in tertiary care hospitals. POD (*n* = 78) and non-delirium groups (*n* = 360) were compared and analyzed for each index in the perioperative period. A prediction model was established using multifactorial logistic regression, and 30 patients' perioperative data were collected for model validation.

**Results:** Eight predictors were included in this study: smoking, diabetes, previous cardiovascular surgery, ejection fraction (EF), time to aortic block, acute kidney injury, low cardiac output syndrome, and pulmonary complications. The area under the receiver operating characteristic (ROC) curve of the constructed prediction model was 0.98 ± 0.005, and the Youden index was 0.91. The validation results showed 97% sensitivity, 100% specificity, and 93% accuracy. The expression of the model was Z = Smoking assignment^*^ – 2.807 – 6.009^*^Diabetes assignment – 2.994^*^Previous cardiovascular surgery assignment – 0.129^*^Ejection fraction assignment + 0.071^*^Brain perfusion time assignment – 2.583^*^Acute kidney injury assignment – 2.916^*^Low cardiac output syndrome assignment – 3.461^*^Pulmonary related complications assignment + 20.576.

**Conclusion:** The construction of an effective prediction model for the risk of delirium in patients after type A aortic stratification can help identify patients at high risk of POD early. It also provides a reference for healthcare professionals in the prevention and care of these patients.

## Background

Aortic dissection (AD) is caused by the tearing of the intimal layer of the aorta or bleeding within the aortic wall, where blood flows between the layers of the aortic wall, separating the layers of the same (entrapment) (1). It is a common condition associated with high mortality (2). Type A aortic dissection involves the ascending aorta and is the most severe type; related studies (3) have shown that the morbidity and mortality rates of patients with type A aortic dissection increase by 1% per hour for 2 days after the onset of the disease. In addition, the morbidity and mortality rate of patients treated non-operatively is up to 74% within 2 weeks, and surgery remains the main treatment for type A aortic dissection (4). However, there are many potential complications after surgery (5), including delirium, which is known to be common after cardiac surgery, with an incidence of 11–46% (6).

According to the Diagnostic and Statistical Manual of Mental Disorders, Fifth Edition (DSM-5) (7) or the International Statistical Classification of Diseases and Related Health Problems, Tenth Edition (ICD 10) (8), delirium is an acute fluctuating change in mental status with reduced consciousness. POD is an acute fluctuating change in the mental state with impaired consciousness and attention. POD is characterized by inattention and fluctuations in consciousness that occur within 30 days after surgery (9, 10) and is associated with high morbidity and mortality, with a prevalence of up to 72% (11). Its incidence depends on risk factors associated with the perioperative period (12). The occurrence of POD prolongs the total length of hospital stay, increases intensive care unit (ICU) morbidity and mortality two to fourfold (13), and significantly increases the level of care dependency in patients who develop POD after discharge (14). This limits their activities for up to 12 months (15) and imposes a huge burden on the individual and society. Here, we investigated the risk factors for POD in patients with aortic dissection and constructed a prediction model to predict such risks.

## Methods

### Study Design, Participants, and Setting

This was a retrospective cohort study with convenience sampling and a randomized controlled trial. Patients who met the inclusion and exclusion criteria in several hospitals in Jiangsu Province from June 2019 to April 2020 were selected. Inclusion criteria: (1) age ≥ 18 years, (2) type A aortic dissection treated with open-heart surgery, (3) no psychiatric or family history, and (4) no previous history of cognitive impairment or stroke. Exclusion criteria: (1) patients with delirium before admission, (2) patients who were in a coma or deep sedation (RASS score ≤ −3) continuously after surgery, (3) patients who were lost to follow-up, patients with incomplete data, and patients with definite cerebrovascular accidents on postoperative computed tomography (CT), (4) patients who stopped treatment unplanned, patients who died, and patients who stopped participation in the study during treatment, and (5) patients who had a second open-heart surgery because of entrapment. This study was approved by the Ethics Committee.

### Measures

#### Delirium

Delirium was assessed during the ICU treatment phase using the Confusion Assessment Method for the ICU (CAM-ICU), which is recommended by the Clinical Practice Guidelines for the Management of Pain, Agitation, and Delirium in Adult Patients in the ICU (16). This method is highly sensitive and specific. Patients were transferred to the ward treatment phase using the 3-min CAM (3D-CAM). The CAM included four aspects: (1) acute alterations and fluctuations in consciousness, (2) inattention, (3) disorganized thinking, and (4) altered clarity of consciousness. Delirium in a patient was determined by the presence of (1) and (2), plus one of (3) or (4) (15).

Sedation levels were assessed using the rating sedation scale (RASS), which allows health professionals to assess the level of bedside sedation or agitation after sedation infusion is stopped (17). Patients were defined as unconscious if they responded to pain/physical stimuli but did not open their eyes (RASS score of −4). A RASS score of −5 indicated that the patient did not respond to physical or verbal stimuli. If the patient had a negative score (> −3 to +4), he or she could be screened for delirium using the CAM-ICU diagnostic tool (18).

The risk factors involved in this study were collected by the investigator on the first day immediately after surgery, and the patients were excluded using the RASS score to identify their coma status. The assessment was stopped if the patient developed POD and continued if the patient did not develop POD until discharge.

### Data Analysis

R 3.6.3 and SPSS 25.0 were used for the statistical analysis of the data. Count data were expressed as frequencies and percentages, and a χ^2^ test was used for comparison between groups. Measurement data obeying normal distribution were expressed as x ± s, and a t-test was used for comparison between groups. Non-normally distributed measurement data were described using medians and quartiles; a rank-sum test was used. The least absolute shrinkage and selection operator (LASSO) regression was used to screen and compress the predictors, and logistic stepwise regression was applied to determine the predictors of POD. The alpha level of the entry model was taken to be 0.05 and that of the exit model was 0.1. The odds ratio (OR) values and 95% confidence intervals (CIs) were used to express the independent predictive ability of the predictors. The area under the receiver operating characteristic (ROC) curve was used to judge the discriminatory ability of the model and to evaluate the ability of the risk factors to predict the occurrence of POD. *p* < 0.05 was considered a statistically significant difference.

## Results

### Participant Characteristics

From June 2019 to April 2020, 469 patients met the inclusion criterion of the surgical treatment of type A aortic dissection; however, 31 patients were excluded. These comprised 14 deaths, 14 discharges against medical advice, and 3 cases with missing data. A total of 438 cases were statistically analyzed: 315 (71.92%) were men and 123 (28.08%) were women. All were within the age range of 18–86 (57.89 ± 12.41). Delirium occurred in 78 cases, with an incidence rate of 17.8%.

### Univariate Analysis of POD

The LASSO regression was used to screen the independent variables to reduce the dimensionality of the data, with 68 starting variables and 17 final variables. Patients in the delirium group were analyzed for risk factors associated with the non-delirium group, and the results showed statistically significant differences (*p* < 0.05) in smoking, diabetes, previous cardiovascular surgery, ejection fraction (EF), EuroScore II, circulatory arrest time, cardio-pulmonary bypass (CPB) time, cross-clamp time, postoperative/cryoprecipitation, acute kidney injury, low cardiac output syndrome, and pulmonary complications (Table 1).

**Table 1:** Univariate analysis of patients with type A aortic dissection (*n* = 438).

**Variable**	**OR**	**95% CI**	***p***
Smoking	0.114	0.064–0.202	<0.001
Diabetes	0.02	0.01–0.04	<0.001
Cardiopulmonary resuscitation within 1 h before surgery	0	0	1
Previous cardiovascular surgery	0.314	0.118–0.839	0.021
EF (%)	0.933	0.904–0.962	<0.001
EuroScore II(%)	1.081	1.044–1.119	<0.001
Circulatory arrest time (min)	1.027	1.015–1.039	<0.001
Assisted circulation time (min)	1.011	1.001–1.021	0.031
Aortic block time (min)	1.059	1.041–1.078	<0.001
Intraoperative blood/cryoprecipitation	1.002	0.996–1.008	0.551
Postoperative/cryoprecipitation	1.282	1.194–1.376	<0.001
Restraint	0	0	0.992
Cumulative postoperative drainage flow (ml)	1	1	0.11
Acute kidney injury	0.087	0.039–0.196	<0.001
Low cardiac output syndrome	0.067	0.021–0.217	<0.001
Lung-related complications	0.039	0.02–0.076	<0.001
Surgical site related infection	0	0	0.999

### Multifactorial Analysis of POD

The statistically significant variables in the univariate analysis were included in the multivariate analysis, and the results showed that smoking, diabetes, previous cardiovascular surgery, EF, aortic block time, acute kidney injury, low cardiac output syndrome, and pulmonary complications independently influenced POD (*p* < 0.05) (Table 2). The final expression of the formula model was Z = smoking assignment^*^ −2.807 – 6.009^*^diabetes assignment – 2.994^*^previous cardiovascular surgery assignment – 0.129^*^ejection fraction assignment + 0.071^*^aortic block time assignment – 2.583^*^acute kidney injury assignment – 2.916^*^low cardiac output syndrome assignment – 3.461^*^lung-related complications assignment + 20.576 (Tables 3, 4).

**Table 2:** Multifactorial analysis of patients with type A aortic dissection (*n* = 438).

**Variable**	**OR**	**95% CI**	***p***
Smoking	0.058	0.016–0.217	<0.001
Diabetes	0.003	0.000–0.019	<0.001
Previous cardiovascular surgery	0.064	0.006–0.709	0.025
EF (%)	0.871	0.804–0.943	0.001
Aortic block time (min)	1.073	1.015–1.135	0.013
Acute kidney injury	0.083	0.009–0.775	0.029
Low cardiac output syndrome	0.039	0.003–0.495	0.012
Pulmonary complications	0.035	0.007–0.185	<0.001

**Table 3:** Logistic regression modeling (*n* = 438).

**Variable**	**β**	**SE**	**Wald**	***p***	**OR**	**95% CI**
Smoking	−2.807	0.641	19.155	0	0.06	0.017–0.212
Diabetes	−6.009	0.941	40.738	0	0.002	0–0.016
Previous cardiovascular surgery	−2.994	1.118	7.167	0.007	0.05	0.006–0.448
EF (%)	−0.129	0.039	10.924	0.001	0.879	0.815–0.949
Aortic block time (min)	0.071	0.021	11.261	0.001	1.073	1.03–1.119
Acute kidney injury	−2.583	1.173	4.851	0.028	0.076	0.008–0.752
Low cardiac output syndrome	−2.916	1.207	5.832	0.016	0.054	0.005–0.577
Pulmonary complications	−3.461	0.75	21.28	0	0.031	0.007–0.137
Constant	20.576	3.946	27.196	0	863424309.8	

**Table 4:** Assignment of factors influencing POD.

**Variable**	**Assignment**
Smoking	0 = No, 1 = Yes
Diabetes	0 = No, 1 = Yes
Previous cardiovascular surgery	0 = No, 1 = Yes
EF (%)	According to the actual score
Aortic block time (min)	According to the actual score
Acute kidney injury	0 = No, 1 = Yes
Low cardiac output syndrome	0 = No, 1 = Yes
Pulmonary complications	0 = No, 1 = Yes

### Modeling

**Figure 1:**
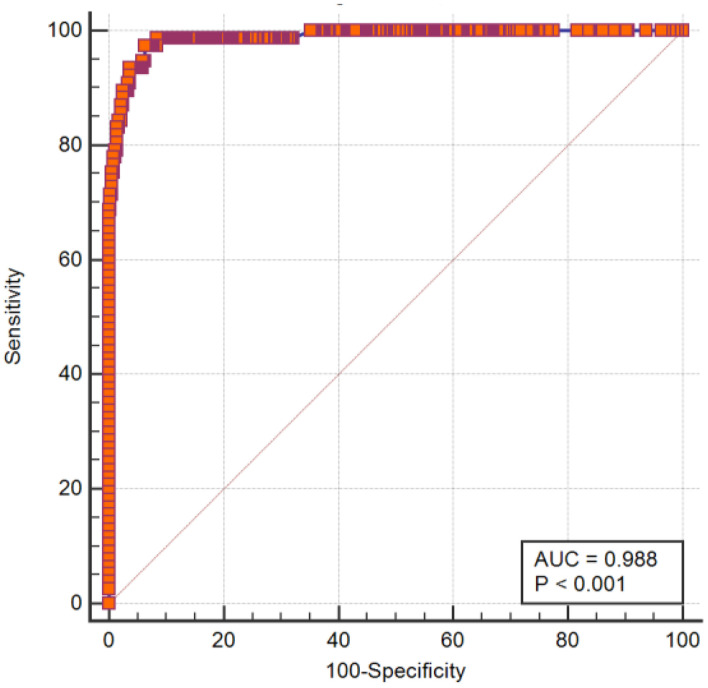
ROC curve of the postoperative delirium prediction model for type A aortic dissection.

Logistic regression models containing the aforementioned eight independent variables were developed using logit (P/1–P) as the dependent variable. A patient's score was calculated using the formula of the prediction model, and the ROC curve was used to test the fitting effect between the patient's model score and the occurrence of delirium in the patient (Figure 1). The maximum value of the Youden index was used as the best critical value of the prediction model. The final measured area under the ROC curve was 0.98 ± 0.005, with a 95% CI of 0.973–0.996. The Youden index of the ROC curve was 0.91; the model sensitivity was 0.97, and the specificity was 0.93 (Figure 1).

### Model Validation

The data of 30 patients undergoing surgical treatment for type A aortic dissection in a tertiary care hospital from May 2020 to June 2020 were selected for model validation. There were 17 men and 13 women, within the age range of 33–85 (60.43 ± 12.89). According to the formula of this prediction model, patients were considered to develop delirium when Z ≥ 0.91. The model predicted that delirium would occur in 3 patients and would not occur in 27 patients. The actual results showed that delirium occurred in 5 patients and did not occur in 25 patients. The sensitivity of the model was 97%, the specificity was 100%, and the accuracy was 93% when the predicted results were compared with the actual results.

## Discussion

Owing to the specificity of the pathogenesis of type A aortic dissection, aggressiveness of the disease, and complexity of the treatment modality (19), delirium is a common and serious neurological complication in patients after surgery (20). Related studies have shown that the incidence of POD after type A aortic dissection ranges from 31.1 to 41.6% (21, 22). POD affects patient prognosis (23), and a model that comprises independent influencing factors with good predictive ability can identify and screen high-risk patients. For the inclusion of variables in this study, recent studies on risk factors for POD were comprehensively analyzed (24, 25), and the final variables included were available in the medical and nursing cases of patients with the characteristics of the study population.

### Patient Factors: Patients With a History of Smoking Are Prone to POD

Smoking and smoking cessation are potentially modifiable risk factors for POD. Certain studies (26, 27) have shown that smokers exhibit severe agitation, sleep disturbances, mood swings, and manifestations associated with delirium. In addition, it was observed that the onset of delirium is associated with a disruption in specific neurotransmitter activity. Patients experience withdrawal symptoms after admission as treatment is initiated and smoking is discontinued. It was proposed that changes in dopamine can explain the different symptoms of delirium and that different levels of dopamine in the body cause different subtypes of delirium (28, 29). During nicotine withdrawal, there is a decrease in voxel dopamine activity. However, an increase in the dopamine release from the prefrontal cortex, which may play an important role in the regulation of anxiety in patients, can elucidate the high prevalence of delirium in patients with nicotine withdrawal, considering the role of the prefrontal cortex in delirium manifestation (30). Recent studies suggest that nicotine withdrawal and delirium share common pathophysiological mechanisms that are mediated by changes in various neurotransmitter systems, including dopamine, opioids, and cholinergic systems. It was also suggested that early identification and management of withdrawal symptoms in patients can improve sleep-wake disturbances and emotional instability, thereby preventing delirium (31–34). Therefore, caregivers should have detailed knowledge of the patient's smoking history, cessation, and possible symptoms and reactions to nicotine withdrawal for timely assessment and prevention.

### Disease Factors: Patients With Diabetes, Previous History of Cardiovascular Surgery, and Low EF Are Prone to POD

Studies have shown a correlation between diabetes and a 1.84-fold increase in the incidence of POD (35–37). Patients with diabetes typically exhibit greater cerebral and hippocampal atrophy (38), cerebral microvascular damage (39), and macrovascular damage (40), compared to those without diabetes. In addition, impaired glucose metabolism exerts important effects on the brain and is a predisposing factor to cognitive impairment (41). Recent studies on epidemiology do not reveal a definite causal relationship; however, evidence suggests (42) the neurotoxic effects of sustained hyperglycemia, which ultimately damages neurons and leads to loss of function. Therefore, nursing staff should understand the patient's history, pay close attention to the patient's blood glucose fluctuations, and cooperate with the physician to promptly improve the patient's blood glucose abnormalities.

Patients with a previous history of cardiovascular surgery are susceptible to POD because of a combination of cardiovascular surgery modalities and previous perioperative risk factors.

Reduced EF causes a reduction in the blood supply to body tissues and organs, which in turn triggers edema and pulmonary stasis due to nutritional deficiencies (43), leading to heart failure. Iwata et al. (44) showed that the degree of delirium correlates with the severity of heart failure, and the development of delirium during hospitalization was associated with short-term and long-term mortality in patients, although this can be predicted by the degree of heart failure.

Cerebral perfusion is dependent on cardiac output, arterial pulsation intensity, cerebral artery patency, cerebral autoregulation, cerebellar vascular patency, and venous patency. If these parameters change, cognition can be affected. Thus, cardiac pathology affecting cerebral hemodynamics may affect cellular function before irreversible structural alterations (45). Decreased cardiac function, which affects the connection between the heart and brain, may lead to acute cognitive impairment, and consequently, POD.

Therefore, nursing staff should closely monitor important reference indicators of cardiac function. In addition, the vital signs of patients with low preoperative EF and poor cardiac function should be closely monitored to obtain relevant examination results. These patients should follow correct medical advice and undergo psychological care and active monitoring to prevent POD.

### Therapeutic Factors: Patients With Long Intraoperative Aortic Block, Acute Kidney Injury in the Perioperative Period, Low Cardiac Output Syndrome, and Pulmonary Complications Are Prone to POD

Extended operative and aortic block times were shown to be predisposing factors for POD (46–48). The aortic block time is an independent risk factor for POD after cardiac surgery. The correlation between the aortic block time and POD has been demonstrated in related studies (49). Andrejaitiene and Sirvinskas (50) reported that the incidence of POD increased eightfold for each minute of an extended aortic block time. This is because long aortic block times induce a risk of inadequate cerebral perfusion in patients, which aggravates brain cell ischemia and hypoxia, causing impaired nerve cell function and affecting the patient's postoperative cognitive function, leading to POD. Nursing staff should understand patients' intraoperative-related passages, identify relevant risk factors, and raise awareness early.

Acute kidney injury (AKI) is commonly observed in patients undergoing cardiac surgery and in the perioperative period of critically ill patients. It can increase morbidity and mortality by affecting distal organ function (51). Improving Global Outcomes (KDIGO) criteria for AKI staging (52), definition of AKI: an increase in serum creatinine by ≥0.3 mg/dl (≥26.5 μmol/l) within 48 h, an increase in serum creatinine to ≥1.5–1.9 times baseline levels, which is known or presumed to have occurred within the prior 7 days and urine volume <0.5 ml/kg/h for 6 h. Numerous studies have shown that acute kidney injury significantly affects the function of extrarenal organs, including the brain (53). Siew et al. (54) reported that acute kidney injury was an independent risk factor for the development of delirium or the diagnosis of coma during critical illnesses. Grigoriieyev et al. (53) confirmed the effects of acute kidney injury on distal organs, verifying that such injuries may lead to a strong inflammatory response with different effects in the brain and lungs. During acute kidney injuries, both organs may interact through multiple mechanisms, such as the amplification of cytokine-induced damage, leukocyte extravasation, sodium and potassium dysregulation, oxidative stress, and use of water channels (55). The occurrence of acute kidney injury in postoperative cardiac patients, regardless of their stage, is associated with an increased incidence of POD (56). Nursing staff should closely monitor the patient's intake and output, pay attention to the patient's laboratory test results, and correctly cooperate with continuous renal replacement therapy (CRRT) and other related treatments.

Certain patients suffer from perioperative low cardiac output syndrome; however, the brain depends on a continuous and adequate blood supply, and disruption of cerebral blood flow can lead to cerebral dysfunction and death (57). Cardiogenic dementia (58) was proposed early in the 1970s, and the concept of cardiocerebral codependency was formed. Ample evidence suggests that adequate cerebral perfusion is a prerequisite for maintaining normal cognitive function (59). It is common for patients to experience a decline in cardiac function, cognitive impairment, and consequently, delirium (60). Nursing staff should closely monitor patients for signs, such as heart rate, blood pressure, and peripheral circulation, with timely feedback and dynamic follow-ups.

The definition of postoperative pulmonary complication (PPC) varies widely, leading to large variation in reported frequency, and is usually dependent upon a set of criteria which may include signs such as chest X-ray findings, pyrexia and positive sputum microbiology. The Melbourne Group Scale (MGS) (61) was used in this study to assess the occurrence of PPC in patients: Temperature>38°C, White cell count >11.2 or respiratory, antibiotics, Physician diagnosis of pneumonia or chest infection, Chest X-ray report of atelectasis/consolidation, Production of purulent (yellow/green), sputum differing from preoperative Positive signs on sputum microbiology, SpO_2_ < 90% on room air and Re-admission to or prolonged stay (over 36 h) on the intensive care unit/high dependency unit for respiratory problems PPC = four or more positive variables. Studies have shown multiple correlations between pulmonary complications and POD, which are mutually causal (62). Patients with pulmonary complications after aortic dissection surgery are prone to hypoxemia, which causes cerebral hypoperfusion and leads to neuroinflammation, as a cause of POD (63). Certain studies have shown that the risk factors and consequences associated with hypoxemia are closely related to delirium; therefore, delirium and hypoxemia are related (63). Considering the severity of pulmonary complications and complex treatment protocols, patients who develop pulmonary complications usually have long hospital stays and are prone to ICU delirium (64). In a study of acute respiratory distress syndrome (ARDS) survivors, the degree of cognitive impairment in these survivors correlated with the severity of hypoxemia. They were observed to have deficits in memory, attention, concentration, and recollection (65). Patients who develop pulmonary complications are at greater risk of POD, resulting from a combination of hypoxemia and inflammation, drug exposure, and prolonged ICU and hospital stay (66).

## Conclusion and Implications for Future Studies

Delirium is a common complication in postoperative patients with type A aortic dissection, and its occurrence is influenced by multiple factors, with nurses playing a crucial role in the prevention phase. Here, a risk prediction model with eight factors was developed to help nurses identify patients at high risk of POD early and execute targeted interventions to reduce the incidence of POD and improve patient prognoses.

Combined with the characteristics of the treatment of type A aortic dissection, patients mostly undergo emergency surgery; therefore, this model performs risk assessment after the surgery. This assessment can be completed based on the patient's surgery records, relevant examination results, and course of the disease records. When the patient's score was greater than or equal to 0.91, it was considered that the patient would probably develop POD and should receive attention from nursing staff in the form of interventions in cooperation with doctors. When the patient's risk assessment was close to the critical value, it was considered that the patient had a certain risk for POD and close attention should be paid to such a patient to prevent POD. When the patient's condition changed during treatment, it was evaluated using a dynamic assessment to minimize the risk of POD.

Compared with previous studies, this study did not stop at the first treatment stage for patients but attempted to include data for the whole perioperative period of patients before, during, and after surgery (including ICU and ward) to avoid missing relevant factors. An evidence-based analysis of relevant risk factors was used; it elucidated gaps in certain studies related to the model for POD in type A aortic dissection. The model involved patient-related assessment data that were simple and easy to obtain, providing a reference for the prevention and treatment of clinical delirium. A limitation of this study is that although the data sources were obtained from multiple hospitals, the sample sizes used in the analysis and that of the model validation were small, and the relevant conclusions require further validation.

## Data Availability Statement

The raw data supporting the conclusions of this article will be made available by the authors, without undue reservation.

## Ethics Statement

The studies involving human participants were reviewed and approved by Ethics Committee of Nanjing First Hospital. Written informed consent for participation was not required for this study in accordance with the national legislation and the institutional requirements.

## Author Contributions

JFH conceived, designed, and implemented the study, collected and analyzed the data, and wrote the article. QL critically reviewed the content of articles and assisted with data collection. YHC was the grantee of the grant program, provided research funding, also gave administrative support and critically reviewed the content of articles. All authors contributed to the article and approved the submitted version.

## Funding

This study was funded by Government of Jiangsu Province, award number: ZKX1803 (grant recipient: YC).

## Conflict of Interest

The authors declare that the research was conducted in the absence of any commercial or financial relationships that could be construed as a potential conflict of interest.

## Publisher's Note

All claims expressed in this article are solely those of the authors and do not necessarily represent those of their affiliated organizations, or those of the publisher, the editors and the reviewers. Any product that may be evaluated in this article, or claim that may be made by its manufacturer, is not guaranteed or endorsed by the publisher.
